# Autophagy activity and expression pattern of autophagy-related markers in the podocytes of patients with lupus nephritis: association with pathological classification

**DOI:** 10.1080/0886022X.2019.1598432

**Published:** 2019-04-24

**Authors:** Juan Jin, Qiudi Tu, Jianguang Gong, Li Zhao, Shikai Liang, Qiang He

**Affiliations:** Department of Nephrology, Zhejiang Provincial People’s Hospital, Hangzhou, China

**Keywords:** Autophagy, podocyte, beclin-1, LC3, Atg7, ULK1

## Abstract

**Objective:** To identify the significance of autophagy in lupus nephritis (LN). **Methods:** The number of autophagosomes in podocytes was counted and the expression of multiple molecular markers associated with autophagy was evaluated in LN specimens: in renal biopsy specimens from 90 patients with LN and 15 healthy controls, autophagosomes in podocytes were counted using transmission electron microscopy and the expression levels of four autophage related proteins including Beclin-1, microtubule-associated protein light chain 3 (LC3), autophagy-related gene 7 (Atg7), and UNC-51-like kinase 1 (ULK1) were measured using immunohistochemistry. **Results:** The number of autophagosomes in patients with LN types III, IV, and combined V–IV type were significantly higher than in controls (*p* < 0.0001; *p* < 0.0001; *p* = 0.009, respectively). However, the autophagosomes numbers in patients with II and V types LN were significantly lower than controls (both *p* < 0.0001). Various levels of marker expression were identified, and they correlated significantly with LN pathology classifications. Moreover, the percentage of marker expression in LN types III, IV and V-IV were significantly higher than controls (*p* < 0.05), while that in types II and V were lower than controls, although the difference for LC3 and ULK1 was not statistically significant. **Conclusions:** Autophagy activity and expression pattern of autophagy-related markers in podocytes were significantly positively correlated with LN of types III, IV, and V–IV, but negatively correlated with II and V types. Therefore autophagy could be a useful predictor of LN pathology type, and be informative and helpful in the development of treatment strategies in clinical settings.

## Introduction

Lupus nephritis (LN) is one of the most common characteristics observed in patients with systemic lupus erythomatosus (SLE). The different renal patterns of injury, including mesangial, endothelial, epithelial (podocyte), tubulointerstitial and vascular involvement, have been associated with different pathogeneses, clinical presentations, therapeutic responses and outcomes in LN patients [1]. The LN classification defined in 2003 International Society of Nephrology and Renal Pathology Society (ISN/RPS) has been widely used internationally [2], despite of some controversies, which include questions about tubulointerstitial involvement and podocyte damage.

Podocytes are highly specialized epithelial cells which line the urinary surface of the glomerular capillary tuft in the kidney, and are involved in the ultrafiltration of blood. To maintain renal filtration function, podocytes usually get together with glomerular endothelial cells and glomerular basement membrane and they jointly resist the high capillary hydrostaic pressure. Moreover, podocytes are not only an important part of the mechanical barrier and charge barrier, but also secrete soluble factors to regulate other types of glomerular cells. Hence, impairment of any of these functions following podocyte injury results in proteinuria and renal failure. Several lines of evidence have confirmed the role of podocyte injury in the pathogenesis of LN [3,4]. Chronic damaging stimuli, such as infection, metabolic factors, and haemodynamic abnormalities, can induce oxidative stress in podocytes, leading to the foot-process effacement of podocytes and eventually the loss of podocytes. Prolonged podocyte injury further leads to glomerulosclerosis and the progression of kidney disease [5].

Autophagy is the cellular process that engulfs, digests and recycles long-lived or aggregated proteins, defective organelles, and various soluble molecules, in order to balance cellular metabolism [6]. Autophagy, hallmarked by the formation of double membrane-bound organelles known as autophagosomes, is the lysosome-dependent pathway for protein degradation, and this process is stimulated and activated in response to metabolic stresses, such as cancer, neurodegeneration, and certain kidney diseases [7–10]. Podocytes normally display the abnormal high level of constitutive autophagy [9]. Autophagy may play a vital role in mouse podocyte differentiation and alleviate the podocyte injury from puromycin aminonucleoside (PAN) [11]. Podocyte injury was associated with changes in autophagy levels, and that rapamycin can activate autophagy in the podocytes induced by PAN and reduce podocyte injury [12].

In the current study, the detailed electron microscopic (EM) analysis of autophagosomes in podocytes and the evaluation of the expression of multiple components in the autophagy pathway, including Beclin-1, microtubule-associated protein light chain 3 (LC3), UNC-51-like kinase 1 (ULK1), and autophagy-related gene 7 (Atg7), were performed for LN patients, in order to assess the potential associations between podocyte autophagy activity and pathological features of LN.

## Materials and methods

### Patient selection and renal biopsies

All LN patients were diagnosed from the results of renal biopsies carried out at the Department of Nephrology, Zhejiang Provincial People’s Hospital, Zhejiang, China. The patients were selected using the ISN/RPS 2003 classification of LN [13]. Exclusion criteria were symptoms of obesity, diabetes mellitus, HBV infection, hepatitis or malignant tumors and patients undergoing continuous renal replacement therapy (CRRT). Control patients (with renal carcinoma) had no clinical features of kidney dysfunction, and their glomeruli were pathologically normal. A total of 90 LN patients (15 type II; 15 type III; 15 type IV; 15 type V; 15 type V–III; and 15 type V–IV) and 15 control patients were enrolled in this study. Podocytes, and autophagosome numbers within the podocytes were analyzed using EM analysis, according to the previous described methods [13–16]. All protocols concerning the use of patient samples in this study were approved by the Human Subjects Committee of Zhejiang Provincial People’s Hospital. Informed written consents were obtained from all donors.

### Reagents and antibodies

Primary antibodies anti-Beclin1 and anti-LC3 (catalogue numbers: GTX113039 and GTX48634 respectively; GeneTex Inc., Irvine, CA), anti-ULK1 (catalogue number: EPR4885(2); Abcam, Cambridge, UK), and anti-Atg7 (catalogue number: DF6130; Affinity Biologicals Inc, Ontario, Canada) were used with corresponding secondary antibodies (Dako, Ely, UK).

### Histological studies of renal specimens

Renal biopsy specimens were examined by light microscopy, direct immunofluorescence (IF) microscopy and EM techniques. All patients were classified according to the ISN/RPS (2003) LN classification [2]. Kidney tissues were fixed in 10% formalin, dehydrated in graded alcohol concentrations, embedded in paraffin, and 2 μm sections were cut from the paraffin blocks. The cut sections were stained with hematoxylin, eosin, and periodic acid–Schiff reagent. All slides were evaluated by the same pathologist, who was blinded to the identities of the specimens, on a Leica 2500 microscope (Leica Biosystems, Milton Keynes, UK) [17]. Blocks of renal cortex tissue (l mm^3^) were fixed in 2.5% glutaraldehyde, followed by postfixation in 2% osmium tetroxide, dehydration in graded acetone and ethanol concentrations, and embedding in epoxy resin (SPI Inc, Westchester, PA). Ultrathin sections (80–90 nm) were stained with uranyl acetate and lead citrate, and then examined with a Tecnai 10 transmission electron microscope (FEI Tecnai, Hillboro, OR) [17]. Autophagosomes in podocytes were identified according to the previously described morphology [16]. The number of autophagosomes was counted in approximately 30 randomly selected podocytes for each set of tissue samples (each set of samples was from five patients), to obtain the average number of autophagosomes per podocyte cell [18].

### Immunohistochemical examination

Immunohistochemiscal staining of renal biopsy was performed as previously described [19]. Briefly, sections from formaldehyde-fixed, paraffin-embedded tissue from 105 specimens were deparaffinised and rehydrated. Antigen retrieval was performed with 0.1 M citrate buffer (pH 6.0) under pressure. After blocking endogenous peroxidase with 3% hydrogen peroxidase, the sections were incubated in 5% normal blocking serum for 20 min. The sections were then incubated with the following antihuman antibodies overnight at 4 °C: Beclin-1, LC3, ULK1, and Atg7. The sections were then incubated for 30 min at room temperature in a proprietary polymer-based secondary antibody (EnVision Systems, Dako, Ely, UK), and then stained with diaminobenzidine (Dako, Ely, UK) for 6 min. The slides were washed with phosphate buffered saline between the steps in the procedure. Sections were then counterstained in Harris’s hematoxylin for 2 min, rinsed in running tap water for 5 min, dehydrated and mounted with hystomount. The above steps were similarly performed for negative control studies despite that the primary monoclonal antibodies mentioned above were not used in the staining stepand replaced by an irrelevant mouse monoclonal antibody.

To ensure the accuracy of the experimental results, all results in immunohistochemical staining were valued by two independent pathologists who were blinded to the clinicopathological data. If there are discordant interpretations, variances were analyzed by a joint review and/or consultation with a third observer familiar with immunohistochemical pathology. According to means described in a previous research [20], the expression levels of Atg7, and ULK1 were scored by the multiplication of the staining intensity (1 = weak; 2 = medium; or 3 = strong) and by the ratio of positive cells (1 = 0–5%; 2 = 6–25%; 3 = 26–75%; or 4 = 75–100%), and a score > 6 was described as strong expression. Beclin 1 and LC3 expression was scored by the summation of the staining intensity (0 = staining; 1 = weak; 2 = medium; or 3 = strong) and the ratio of positive cells (0 = 0–9%; 1 = 10–49%; 2 = 50–100%), and scores of 4 and 5 were considered as strong expression, as formerly described [21].

### Statistical analysis

Statistical analysis was performed using SPSS 19.0 software (IBM, Armonk, NY). Data were expressed as mean values ± standard deviations or median [interquartile range (IQR)] or ratio. Differences between groups were determined by Student’s *t*-tests and Mann-Whitney *U* tests. A *χ*^2^ test or continuity correction *χ*^2^ test or Fisher exact probability test was used to analyze the association between several clinicopathological factors and expression levels of molecular markers. Differences were considered significant when *p* < 0.05.

## Results

### Relationship between LN pathological types and clinical manifestations

The mean age of the 90 patients at the time of renal biopsy was 30.4 ± 10.5 years. The male to female ratio was 1:9. The detailed general clinical and pathological data are not shown. The general clinical data of the 90 LN patients were shown in Table 1. Based on the general clinical data in Table 1 and according to the 2003 ISN/RPS classification system for LN, 15 patients were classified as class as type II, 15 as type III, 15 as type IV, 15 as type, 15 as type V–III, and 15 as type V–IV.

**Table 1.** Relationship between LN pathological types and clinical manifestations.

Clinical data	LN pathological types						Total
	Type II (*n =* 15)	Type III (*n =* 15)	Type IV (*n =* 15)	Type V (*n =* 15)	Type V + III (*n =* 15)	Type V + IV (*n =* 15)	
Gender							
Male	1	1	2	3	0	2	9
Female	14	14	13	12	15	13	81
Age (year)	26.2 ± 9.9	28.4 ± 10.5	28.7 ± 11.5	33.3 ± 10.5	25.40 ± 10.7	33.6 ± 11.9	30.5 ± 10.5
Clinical type							
Occult nephritis	8	0	0	0	0	0	8
Nephritic syndrome	7	13	8	5	1	1	35
Nephrotic syndrome	0	2	6	10	14	10	42
Rapidly progressing glomerulonephritis	0	0	1	0	0	4	5
Total	15	15	15	15	15	15	90

The clinical manifestations of LN of different types were summarized in Tables 2 and 3. In Table 2, it was showed that the levels of proteinuria was generally positively associated with the grades of LN and the manifestations of hypertension, hematuria, macrohematuria, and proteinuria etc., were more significant in type IV; in Table 3, it was showed that for most of the indictors related to autoantibody, their amounts in type IV LN was higher. In summary, type IV LN demonstrated the most significant clinical manifestations.

**Table 2.** Renal manifestations of patients.

	II (*n =* 15)	III (*n =* 15)	IV (*n =* 15)	V (*n =* 15)	V + III (*n =* 15)	V + IV (*n =* 15)
Hypertension	2 (13.3)	4 (26.7)	7 (46.7)	3 (20.0)	4 (26.7)	7 (46.7)
Hematuria	3 (20.0)	9 (60.0)	12 (80.0)	5 (33.3)	10 (66.7)	12 (80.0)
Macrohematuria	0	1 (6.7)	2 (13.3)	0	1 (6.7)	1 (6.7)
Urine protein ≥ 0.5 g/day	11 (73.3)	13 (86.7)	14 (93.3)	14 (93.3)	14 (93.3)	15 (100.0)
sCr ≥1.3 mg/dL	0	1 (6.7)	5 (33.3)	1 (6.7)	2 (13.3)	4 (26.7)

For each data in the table, the number outside the parentheses was the number of the patients positive for the specific manifestation; the number inside the parentheses was the percentage of the patients positive for the specific manifestation in each classification group.

**Table 3.** The serum autoantibody manifestations of patients.

	II (*n =* 15)	III (*n =* 15)	IV (*n =* 15)	V (*n =* 15)	V + III (*n =* 15)	V + IV (*n =* 15)
ANA	14 (93.3)	12 (80.0)	14 (93.3)	11 (73.3)	13 (86.7)	13 (86.7)
A-dsDNA	7 (46.7)	7 (46.7)	10 (66.7)	4 (26.7)	5 (33.3)	9 (60.0)
A-Sm	4 (26.7)	3 (20.0)	4 (26.7)	3 (20.0)	3 (20.0)	3 (20.0)
A-RNP	4 (26.7)	4 (26.7)	4 (26.7)	3 (20.0)	6 (40.0)	4 (26.7)
ANCA	0	1 (6.7)	1 (6.7)	0	0	0
SSA	4 (26.7)	7 (46.7)	5 (33.3)	6 (40.0)	6 (40.0)	6 (40.0)
SSB	1 (6.7)	3 (20.0)	2 (13.3)	2 (13.3)	3 (20.0)	2 (20.0)
Low C3	8 (53.3)	11 (73.3)	12 (80.0)	8 (53.3)	11 (73.3)	13 (86.7)
Low C4	2 (13.3)	7 (46.7)	7 (46.7)	2 (13.3)	3 (20.0)	7 (46.7)

For each data in the table, the number outside the parentheses was the number of the patients positive for the specific manifestation; the number inside the parentheses was the percentage of the patients positive for the specific manifestation in each classification group.

### The podocyte injury of the patients grouped by different pathological classifications of LN

In this study, the data of the podocyte injury for the patients with different types of LN was collected and demonstrated in the form of percentage (Table 4). The data showed that that the podocyte fusion reached high at type II and suddenly dropped to bottom at type III and rose from type IV to type V and reached plateau from type V + III to type V + IV. The mechanism for this pattern was not clear currently and would be one of the aims in the furhter investigation.

**Table 4.** The comparison of the data for the podocyte fusion between different pathological classifications of LN.

The data for the podocyte fusion in different types of LN						
Type	II (*n =* 15)	III (*n =* 15)	IV (*n =* 15)	V (*n =* 15)	V + III (*n =* 15)	V + IV (*n =* 15)
Mean ± SD	57.0 ± 10.8	29.7 ± 7.4	42.0 ± 12.5	60.7 ± 16.8	55.0 ± 11.0	56.3 ± 13.3
Comparison between groups (*p*-value)						
Type	II	III	IV	V	V + III	V + IV
II		0.000	0.002	0.175	0.620	0.881
III			0.003	0.000	0.000	0.000
IV				0.002	0.005	0.005
V					0.284	0.440
V + III						0.767

All date expressed as number (percentage).

### Relationship between the autophagic activity in podocytes and the pathological types of LN

**Figure 1.**
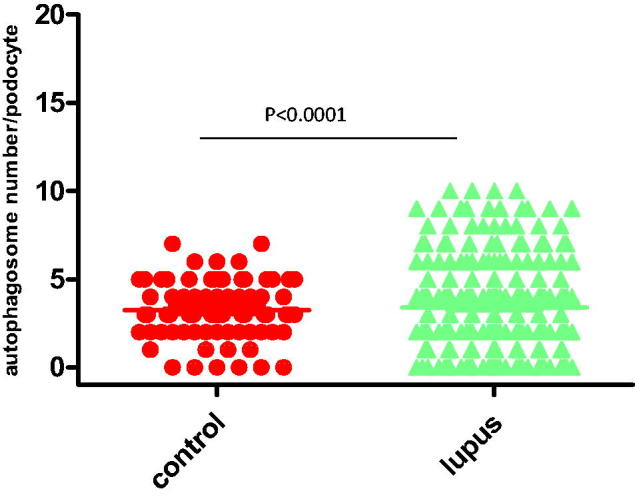
Quantification of podocytes containing autophagosomes in kidney sections from 90 LN patients and 15 healthy controls; the percentage of autophagosome-positive podocytes in LN biopsies was significantly higher than in controls (*p* < 0.0001).

**Figure 2.**
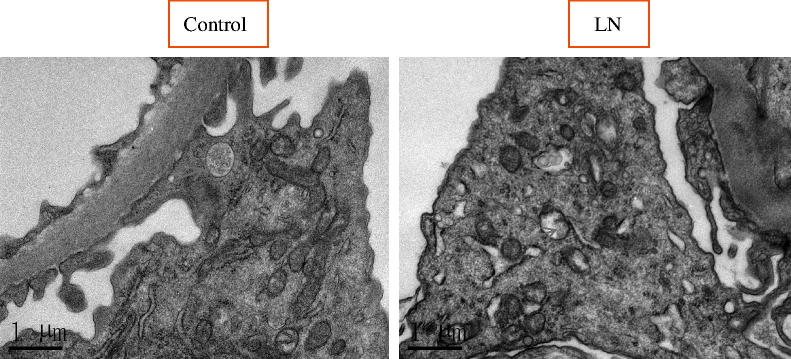
Autophagosomes (arrows) detected by EM in the podocytes of LN patients and controls.

**Figure 3.**
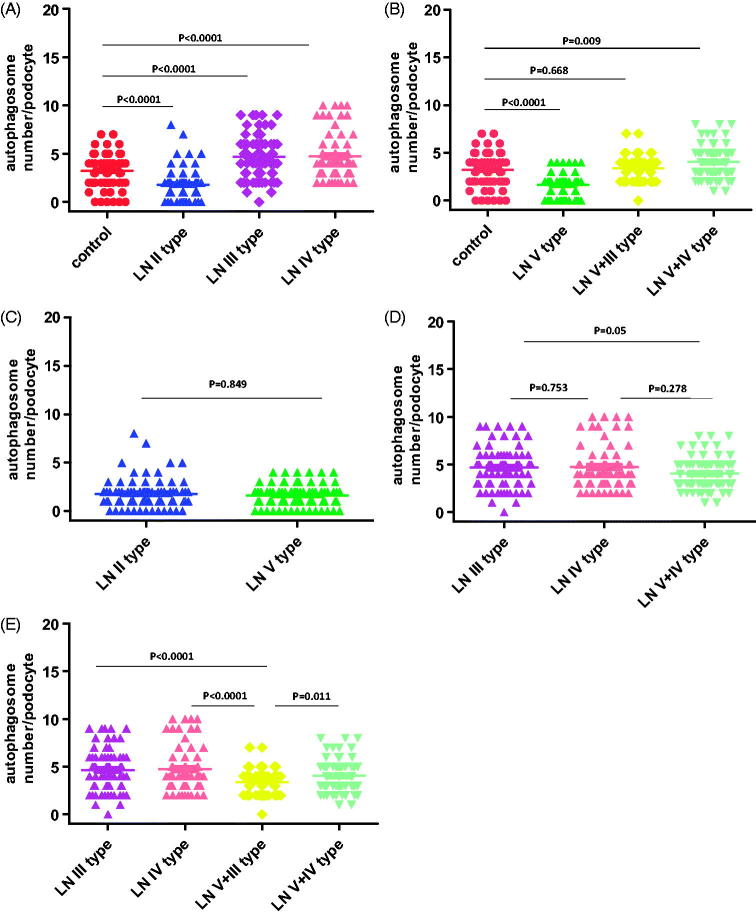
The comparison of the number of autophagosomes in podocytes between the various types of LN.

To study the potential role of autophagic activity in modulating the progression of LN, the number of autophagosomes in podocytes for LN patients and healthy controls were compared using EM analysis (Table 5; Figure 1). The results showed that there was more autophagosomes in podocytes from LN patients than those from healthy controls. Representative findings of EM analyses are shown in Figure 2. Interestingly, the number of autophagosomes in podocytes was significantly different among the various types of LN (Table 5; Figure 3(A,B)): the numbers of autophagosomes in podocytes in type II and type V samples were significantly smaller than that in the controls (*p* < 0.0001; *p* < 0.0001, respectively); however, the numbers of autophagosomes in podocytes in type III, type IV, and type V–IV were significantly larger than that in the controls (*p* < 0.0001; *p* < 0.0001; *p* = 0.009, respectively); moreover the number of autophagosomes in podocytes was bigger in type V-III than in the control group, but the difference did not reach statistical significance; in addition, the difference of the numbers of autophagosomes in podocytes between type II and type V samples did not reach statistical significance either (Figure 3(C)); similarly, among the three groups in which the number of autophagosomes in podocytes were significantly larger than that in controls (types III, IV, and V–IV), the differences among them did not achieve statistical significance (Figure 3(D)); finally, it was interesting to note that the number of autophagosomes in podocytes among types III, V–III, IV, and V–IV were significantly different.

**Table 5.** Autophagosomes in podocytes from LN patients.

Group	Number	Autophagosomes/podocyte
Control	15	3 (2, 5)
LN	90	3 (2, 5)
Type II	15	2 (1, 2)
Type III	15	5 (3, 6)
Type IV	15	4 (3, 5)
Type V	15	2 (1, 2)
Type V–III	15	3 (3, 4)
Type V–IV	15	4 (3, 5)

The data were demonstrated in the form of M(P25, P75).

### The expression levels of multiple autophagic biomarkers in LN specimens

**Figure 4.**
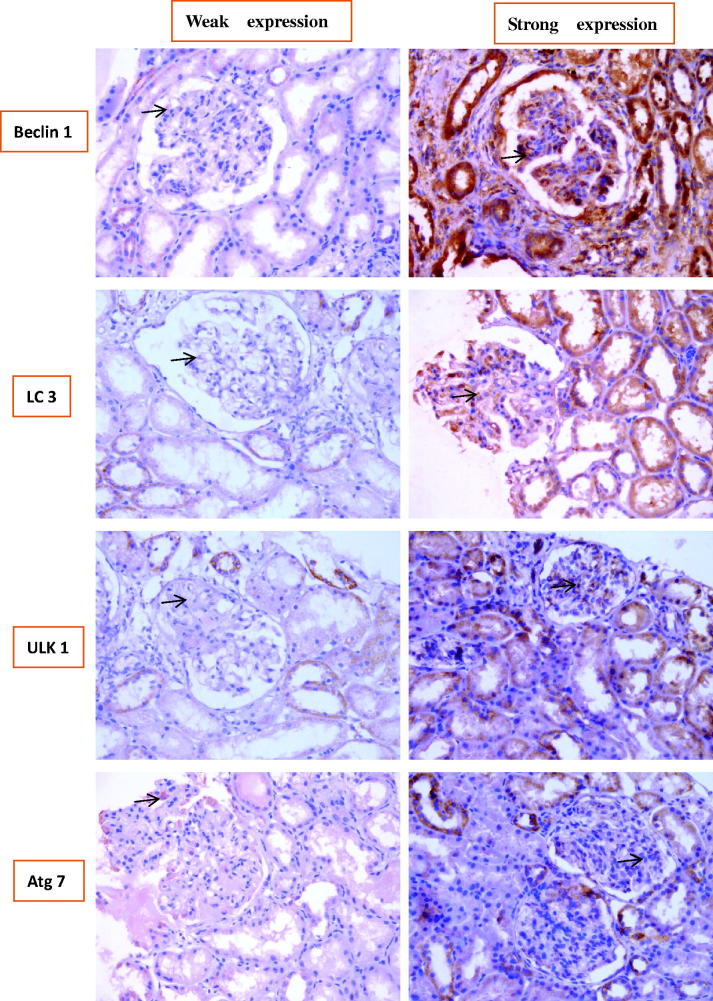
Representative findings on immunohistochemical staining of renal biopsy specimens from patients with LN with antibodies against Beclin1, microtubule-associated protein light chain 3 (LC3), and UNC-51-like kinase 1 (ULK1) autophagy-related gene 7 (Atg7). The arrows pointed to the positive signals.

To validate the above EM results, which idicated that there was higher autophagic activity from LN patients than that from healthy controls in glomeruli, particularly in podocytes, and to find the most efficient interfering points to delay the progress of LN, the expression levels of four autophagic biomarkers (Beclin-1, LC3, ULK1, and ATG7) in renal tissues from LN patients and control specimens were measured. Representative findings on immunohistochemical staining were shown in Figure 4.

Table 6 showed that the correlation between the expression levels of the four autophagic biomarkers and the various types of LN: consistent with the above EM results, the expression levels of the four autophagic biomarkers were significantly different between the various types of LN; expression levels of Beclin-1 and ULK1 in types II V type were significantly lower than those in controls (*p* < 0.05), while expression levels of LC3 and Atg7 in types II–V were also lower than those in controls, although the difference wasnot statistically significant and the expression levels of the four biomarkers in types III, IV, and V–IV type were significantly higher than those in controls (*p* < 0.05); and statistically significant differences were not observed between LN type V–III and controls; most importantly, the expression levels of those four biomarkers were generally strongest in type IV, which was similar to the clinical manifestation data in Tables 2 and 3, which implied that the intervention towards those four biomarker in type IV LN may be most efficient to delay the progress of LN.

**Table 6.** Correlation of biomarkers associated with autophagy in the various LN types.

Variables	Beclin 1 expression (%)			LC3 expression (%)			ULK1 expression (%)			ATG7 expression (%)		
	Weak	Strong	*p*-Value	Weak	Strong	*p*-Value	Weak	Strong	*p*-Value	Weak	Strong	*p*-Value
Control	10 (66.7)	5 (33.3)		11 (73.3)	4 (26.7)		9 (60.0)	6 (40.0)		11 (73.3)	4 (26.7)	
LN II type	14 (93.3)	1 (6.7)	0.042	15 (100.0)	0 (0.0)	0.1000	15 (100.0)	0 (0.0)	0.017	15 (100.0)	0 (0.0)	0.1000
LN III type	3 (20.0)	12 (80.0)	0.025	4 (26.7)	11 (73.3)	0.027	2 (13.3)	13 (86.7)	0.021	3 (20.0)	12 (80.0)	0.009
LN IV type	1 (6.7)	14 (93.3)	0.002	0 (0.0)	15 (100.0)	<0.0001	2 (13.3)	13 (86.7)	0.021	1 (6.7)	14 (93.3)	<0.0001
LN V type	15 (100.0)	0 (0.0)	0.042	15 (100.0)	0 (0.0)	0.1000	15 (100.0)	0 (0.0)	0.017	14 (93.3)	1 (6.7)	0.330
LN V + III type	11 (73.3)	4 (26.7)	1.000	10 (66.7)	3 (20.0)	1.000	12 (80.0)	3 (20.0)	0.427	11 (73.3)	4 (26.7)	1.000
LN V + IV type	3 (20.0)	12 (80.0)	0.025	3 (20.0)	12 (80.0)	0.009	1 (6.7)	14 (93.3)	0.005	2 (13.3)	13 (86.7)	0.003

For each data in the table, the number outside the parentheses was the number of the patients positive for the specific proteins; the number inside the parentheses was the percentage of the patients positive for the specific proteins in each classification group.

## Discussion

The pathogenesis of LN is extremly sophisticated and regulated by multiple factors. Hartleben et al. [9], showed that podocytes occupy a relatively high level of autophagic activity, and that physiological autophagy in podocytes is critical to the maintenance of normal structure and function. In the current study, there was a relatively high level of autophagic activity in podocytes of healthy controls. The present study, in which LN patient samples of podocyte injury were included, showed that autophagic activity in glomeruli, particularly in podocytes, may play a critical role in modulating the progression of LN. In glomeruli from LN patients, autophagosomes were mostly observed in podocytes, consistent with previous findings that podocytes display a relatively high basal level of autophagic activity [9,22,23]. In this study, it was also observed that the autophagic activity in podocytes was significantly different among the various types of LN. In this series, various expression levels of the four autophagy-related markers were detected in the majority of LN tissues. Based on precise evaluation of the expression level of each marker in biopsies, considering the staining area as well as its intensity, all four markers were shown to be significantly correlated with pathological type. The above findings strongly indicated that the molecules involved in autophagy played important roles in the regulation of LN progression.

The final meaning of the clinical and laboratory study was to support the current treatment for specific diseases and the final aim of this study was not the exception. In this study, two pieces of findings were most important and might help the treatment for LN: (1) in type IV LN, the clinical manifestations were most severe (Tables 2 and 3); (2) also in type IV LN, the four studied biomarkers related to the autophage process (Beclin-1, LC3, ULK1, and ATG7) had the highest expression levels. The above two finding implied that the intervention methods targeting those four biomarkers in type IV LN might be most efficient to delay the progress of LN.

In the present study, the podocyte injury of the patients grouped by different pathological classifications of LN was demonstrated by the percentage data for the podocyte with fusion (Table 4) and interestingly, it was found that the popdocyte fusion was high at type II and dramatically low at type III and gradually climbing from type IV to type V and finally flat from type V + III to type V + IV. The mechanism for the above pattern of the podocyte injury needs further investigation.

In conclusion, this study demonstrated that podocyte autophagic activity was a crucial factor in renal injury, and that several biomarkers for autophagic activity may regulate the autophage in podocytes during the early stages of disease. Targeting those biomarkers (inhibiting those biomarkers) may therefore be the potential therapeutic approach to delay the progression of LN, particularly interfering the four biomarkers (Beclin-1, LC3, ULK1, and ATG7) in the type IV LN. So, this study would be informative for the development of more specific treatment strategies.
